# Changes in Microbial Community Structure in Response to Gummosis in Peach Tree Bark

**DOI:** 10.3390/plants11212834

**Published:** 2022-10-25

**Authors:** YoungJae Jo, Da-Ryung Jung, Tae-Hyung Park, Dokyung Lee, Min-Kyu Park, Kyeongmo Lim, Jae-Ho Shin

**Affiliations:** 1 Department of Applied Biosciences, Kyungpook National University, Daegu 41566, Korea; 2 Department of Integrative Biotechnology, Kyungpook National University, Daegu 41566, Korea; 3 NGS Core Facility, Kyungpook National University, Daehak-ro 80, Daegu 41566, Korea

**Keywords:** peach gummosis, microbiome, mycobiome, agroecosystem, plant-microbe interactions

## Abstract

Peach gummosis disease has been identified as a serious challenge in Korean agriculture and has developed to become a major cause of agricultural productivity losses. However, treatments for gummosis have not been systemically established and studies of the microbiome closely related to this plant disease are lacking. Therefore, we analyzed the bacterial and fungal communities in the bark and rhizosphere soil of healthy peach trees and those with gummosis. Through high-throughput sequencing, we obtained unprecedented insights into the bacterial and fungal dynamics of each group, including their diversity and taxonomic classification, as well as network analyses. We found that the presence of gummosis drives a significantly higher alpha diversity in the bark bacterial community. Peach gummosis bark mycobiomes included greater numbers of opportunistic pathogens such as *Ascochyta*, *Botryosphaeria*, *Saccharomyces*, *Nectriaceae_NA*, *Trametes*, and *Valsaceae_NA.* However, the microbiome also included bacteria beneficial to plant growth and the production of polysaccharides—namely, *1174-901-12*, *Catenibacterium*, *Cutibacterium*, *Friedmanniella*, *Methylobacterium-Methylorubrum*, *Pseudomonas*, *Rhodobacter*, and *Sphingomonas*. Furthermore, we confirmed that gummosis induced a more complex structure in the bark microbiome network. We conclude that the findings of this study provide a valuable aid in profiling the overall peach tree microbial ecosystem, which can be utilized to develop precise biomarkers for the early diagnosis of gummosis.

## 1. Introduction

Peaches are the third most cultivated fruit crop in South Korea, making the country one of the leading producers of peaches in the world. The area under peach tree cultivation is approximately 20,000 ha, accounting for 13.2% of the total fruit tree cultivation area in the country. Gummosis, a disease of fruit trees, is characterized by a dark brown resin secreted from the stems or branches of the tree and occurs periodically throughout the cultivation cycle [1]. The disease first occurs in the flowering period (early stage of growth) and is most prevalent during the monsoon. The resin leaks through physical wounds such as gum extrusions on the bark, limbs, and twigs [2,3,4], and continuous resin leakage can seriously affect crop productivity [5].

Although factors such as environmental stress and physical or chemical injuries may partly determine the occurrence or severity of gummosis [6], infection with pathogenic fungi and bacteria appears to be the main cause of the disease. *Botryosphaeria* species—including *B. dothidea*, *B. obtusa*, and *B. rhodian*—are commonly detected in peaches [1,2,4,5,7,8,9,10], and *Pseudomonas syringae*—which causes bacterial gummosis—has been reported [11,12]. Studies in which factors (such as a particular pathogen) are examined in isolation are limited in the sense that they do not describe the complete microbial ecosystem of the host plant. In addition, several studies have shown that resin, besides being a symptom of gummosis, is a means of protecting the plant from insects and pathogens [13,14,15,16]. Accordingly, the elucidation of plant–microbe interactions through microbial community analysis is warranted.

Peach trees are vulnerable to latent pathogenic infections year-round. There are as of yet no effective or approved fungicides for the control of peach gummosis, and although pesticides are used to prevent fruit and leaf diseases, such applications can prove expensive [17,18,19]. Therefore, with the goal of the continuous management of gummosis, it is necessary to discover microbial sources that inhibit the development of the disease and explore the microbial community in various plant environments. This study aimed to reveal differences in the bacterial and fungal communities in the bark endosphere and rhizosphere of healthy peach trees (HP) and peach trees with gummosis (PG). Our analysis of the dynamics of bacterial and fungal community structures following gummosis suggests that there are taxa that may be closely related to gumming syndrome. Our identification of these taxa is expected to aid in the development of biomarkers for gummosis and contribute to a more comprehensive understanding of plant–microbe interactions as they relate to disease.

## 2. Results

### 2.1. Biodiversity in Bark and Soil

**Figure 1:**
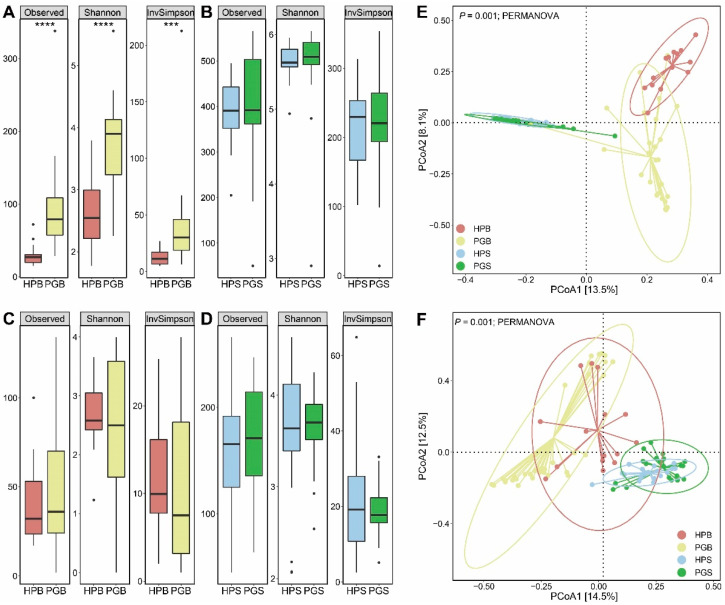
Biodiversity of the microbial community in soil and bark according to gummosis. The box plots of the three indexes (Observation, Shannon, and InvSimpson) represent the microbial alpha diversity values for each group. (**A**) Bacterial alpha diversity between HPB and PGB. (**B**) Bacterial alpha diversity between HPS and PGS. (**C**) Fungal alpha diversity between HPB and PGB. (**D**) Fungal alpha diversity between HPS and PGS. (Mann–Whitney, *** *p* < 0.001, and **** *p* < 0.0001). (**E**,**F**) Beta diversities based on Bray–Curtis dissimilarity reveal significant differences between 4 groups via PCoA. (**E**) Beta diversity of microbiome (PERMANOVA, *p* = 0.001). (**F**) Beta diversity of mycobiome (PERMANOVA, *p* = 0.001).

First, we evaluated the alpha diversity of the microbial community with and without the presence of gummosis. In the case of the Observed, Shannon, and InvSimpson indices, the bacterial diversity of the peach gummosis bark (PGB) was far higher than that of the healthy peach bark (HPB; Mann–Whitney, *p* < 0.0001 for Observed and Shannon, and *p* < 0.001 for InvSimpson, respectively; Figure 1A). Notably, this considerable difference between gummosis-infected trees and healthy trees was only evident in the bark bacteria and not in the soil bacteria, bark fungi, or soil fungi (Mann–Whitney, *p* > 0.05; Figure 1B–D). In the four groups comprising HPB, PGB, HPS (healthy peach soil), and PGS (peach gummosis soil), it was confirmed that the bacterial communities were clustered within each group (PERMANOVA, *p* < 0.001; Figure 1E). Interestingly, this clustering result was also detected in the fungal community (PERMANOVA, *p* < 0.001; Figure 1F). The results of the comparison of bacterial and fungal communities in the bark of healthy and gummosis peach trees (HPB and PGB), as well as the comparison of bacterial and fungal communities in the soil of healthy and gummosis peach trees (HPS and PGS), indicated a significant dissimilarity (PERMANOVA, *p* = 0.001 and *p* = 0.004, respectively, for bark and soil analyses; Supplementary Figure S1A–D). In addition, microbiological dissimilarities based on unweighted UniFrac revealed consistent patterns, although weighted UniFrac demonstrated statistical significance only when comparing HPS to PGS (PERMANOVA, *p* = 0.035; Figure S1E–L).

### 2.2. Extensive Profiling of Taxonomic Composition

**Figure 2:**
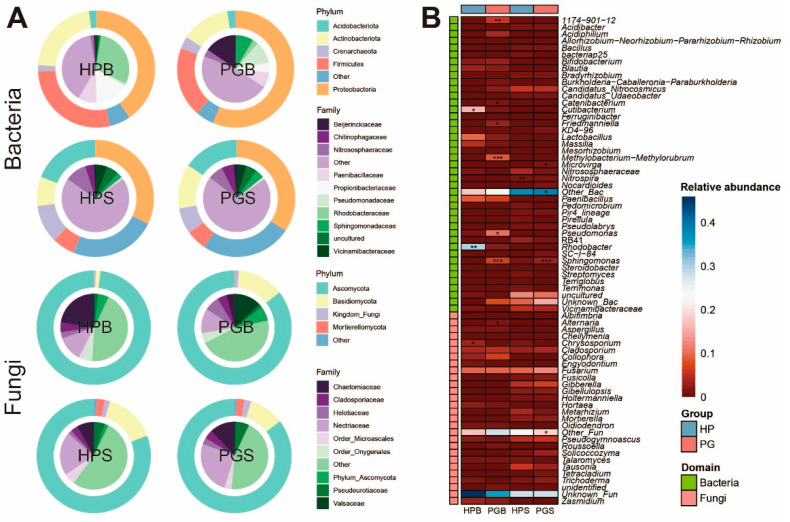
Microbial taxonomic composition of each group. (**A**) Combined donut and pie charts indicate phylum and family, respectively. (**B**) Heatmap showing the difference in microbial abundance at the genus level, either between bark (HPB and PGB) or soil (HPS and PGS). Mann–Whitney U tests were applied to assess statistical significance. * *p* < 0.05, ** *p* < 0.01, *** *p* < 0.001.

We further evaluated the microbial composition of all the groups to understand the dynamics of the microbial community following gummosis. Overall, taxonomic classification was visualized with the top five phyla and 10 families (Figure 2A). Among these phyla, Proteobacteria (40.8%) and Ascomycota (80.5%) dominated the bacterial and fungal communities, respectively. Moreover, we confirmed that the PGB microbiota is extensively different from that of HPB. In particular, Beijerinckiaceae (0.7%) and Rhodobacteraceae (2.7%) were considerably lower in PGB than in HPB. Specific information on the microbial composition at the phylum and family levels is described in Table S1. When the ASV IDs were assigned to the genus level, the HP groups (HPB and HPS) and PG groups (PGB and PGS) showed statistically significant differences. We filtered out the genera that had low prevalence and relative abundance, as described in the Materials and Methods, leaving 43 genera of bacteria and 28 fungal genera. We then found statistical significance in the case of 10 genera (bacteria: *1174-901-12*, *Catenibacterium*, *Cutibacterium*, *Friedmanniella*, *Methylobacterium-Methylorubrum*, *Pseudomonas*, *Rhodobacter*, *and Sphingomonas*. Fungi: *Alternaria* and *Chrysosporium.* Mann–Whitney, * *p* < 0.05, ** *p* < 0.01, *** *p* < 0.001) in the bark, and three genera (bacteria: *Microvirga*, *Nitrospira*, and *Sphingomonas.* Mann–Whitney, *p < 0.05, **p < 0.01, *** *p*< 0.001) in the soil (Figure 2B). Although significant differences were observed, taxa excluded from the heatmap due to low prevalence and relative abundance were displayed in Figure S2. Taken together, this broad analysis of taxonomic classification illustrates that these bark and soil microbiomes are possibly associated with gummosis.

### 2.3. Biomarker-Mining for Gummosis Diagnosis

**Figure 3:**
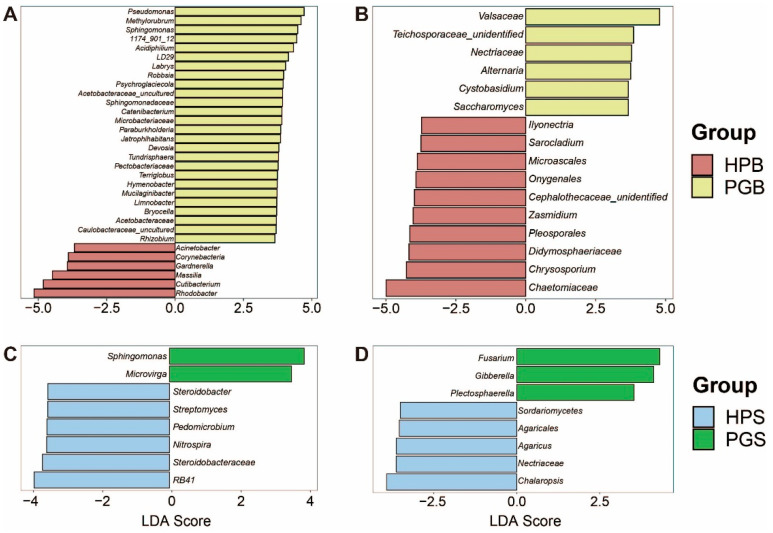
Linear discriminant analysis effect size (LEfSe) analysis shows representative taxa in each group, according to gummosis. Each bar plot represents the result of LEfSe (**A**–**D**). Representative genera of (**A**) bacteria in bark, (**B**) fungi in bark, (**C**) bacteria in soil, and (**D**) fungi in soil.

To verify these genera as potential biomarkers for the early diagnosis of gummosis, we conducted a linear discriminant analysis (LDA) effect size (LEfSe) analysis. The LEfSe results, showing the optimal features to account for group differences by LDA score, indicated genera with LDA scores of 3.5 or higher that were representative of each group. In multiple comparisons, including HPB–PGB and HPS–PGS, numerous taxa were identified to discriminate between individual groups. Specifically, the microbial community in the HPB comprised 16 genera (6 bacteria and 10 fungi), whereas PGB comprised 33 genera (27 bacteria and 6 fungi; Figure 3A,B). Conversely, in the soil microbial communities, only five (two bacteria and three fungi) genera were found in PGS and 11 (six bacteria and five fungi) in HPS (Figure 3C,D). It was confirmed that the representative genera of the bark microbial community—namely *Pseudomonas*, *Methylobacterium-Methylorubrum*, *Sphingomonas*, *1174-901-12*, *Cutibacterium*, *Rhodobacter*, *Alternaria*, and *Chrysosporium*—showed a significant difference in relative abundance as well. These results demonstrate noticeable differences in microbial communities depending on the presence or absence of gummosis; the strong associations seen with gummosis suggest the potential of specific genera in PGB as biomarkers of gummosis.

### 2.4. Network Analysis

**Figure 4:**
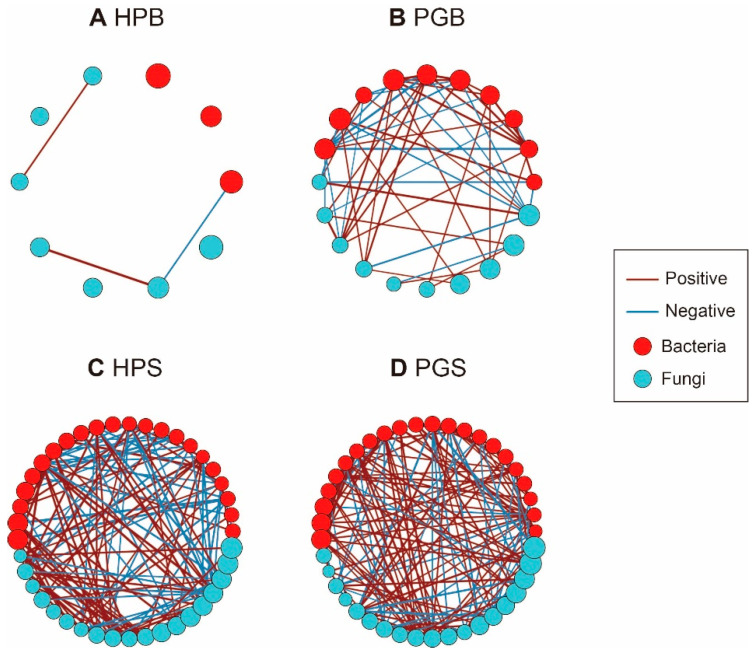
Co-occurrence network analysis of bark and soil microbial community according to gummosis. (**A**) HPB, (**B**) PGB, (**C**) HPS, and (**D**) PGS. Each network structure contains 10, 20, 41, and 41 nodes and 3, 55, 137, and 168 edges, respectively. Nodes and edges indicate microbial genera and significant correlations. The colors of nodes and edges represent the corresponding phyla and the direction of the relationship.

To investigate the interactions between the bacterial and fungal genera within each group, four network communities were generated. Each of the four groups (HPB, PGB, HPS, and PGS) consisted of 10, 20, 41, and 41 nodes and 3, 55, 137, and 168 edges, respectively (Figure 4). Network density (D), defined as the ratio of the number of edges to the number of possible edges, shows how intensively the nodes comprised linkages within their groups. The difference in network density was much larger in the bark group than in the soil group, as HPS and PGS revealed network densities of 0.17 and 0.20 (Figure 4C,D) whereas the corresponding values in HPB and PGB were 0.07 and 0.29, respectively (Figure 4A,B). Its large number of nodes, many edges, and high density suggest that PGB is a more complex microbial ecosystem than HPB. Furthermore, we assessed the pattern of network transitivity (T)—representing the clustering coefficient—and found that the pattern of microbial networks of barks and soil (T = 0, 0.54, 0.54, 0.46 in HPB, PGB, HPS, and PGS, respectively) was parallel to the network density.

## 3. Discussion

Microbiomes are widely perceived as a requisite portion of the crop ecosystem and are strongly associated with plant growth and disease resistance [4,20,21,22]. Although an increasing number of studies have shown that biotic or abiotic stresses perturb the microbiome of various organs in plants [23,24,25,26], standardization of the peach microbial community following gummosis is unclear. In recent years, microbiome studies on peach and gummosis disease have been conducted extensively on a single organ or at the level of a single domain [27,28,29,30,31], but studies of epiphytic and endophytic microbial communities comprised of fungi and bacteria are lacking. To the best of our knowledge, this study is the first in which microbial and fungal communities were investigated in multiple environments such as the bark endosphere and rhizosphere following gummosis. The findings, through amplicon sequencing, enhance the understanding of the bark microbiome response to gummosis and suggest that the bark microbial community (at the level of the genus) is a potential diagnostic of peach gummosis disease.

To date, the diversity of the rhizosphere microflora in relation to plant diseases has been extensively reported through studies on plant–microbial interactions [21,28,30,32,33,34,35,36,37,38]. However, unlike in previous studies, noticeable changes in the soil microbial community following gummosis were not observed. Even though the beta diversity of microbial communities was markedly dissimilar between HPS and PGS in the case of both bacteria and fungi (Figure S1B,D), the equivalent level of alpha diversity and microbial diversity within each group was also examined (Figure 1B,D). In addition, as a result of comparing HPS and PGS, there was no significant difference in the microbial community in soil compared to that in the bark. The alterations in the microbial community caused by gummosis were found to be more sensitive in bark, and this result may suggest a prioritization of approaches for understanding gummosis driving perturbation in microbial ecosystems. Interestingly, we found beneficial bacteria (instead of pathogens) in PGS. Genera with significant differences between HPS and PGS, including *Microvirga*, *Nitrospira*, and *Sphingomonas*, have been reported in healthy soils or as being involved in nitrification. There are numerous reasons why this phenomenon might exist. The rhizosphere may only be slightly impacted by the gummosis disease that emerges in the bark, or it may even be a microbial defense mechanism against plant stress. Numerous previous studies have demonstrated that plants can employ a “cry for help” strategy to enhance their ability to resist stress from illnesses, and this strategy is consistent with our findings [39,40].

The microbial community under gummosis in the bark differed noticeably from that in the soil. First, a comparison of HPB and PGB showed only a significant difference in alpha diversity, indicating which community should be focused on—the rhizosphere or endophytic—to understand microflora changes following gummosis. Alpha diversity indices have been used as indicators of host plant health in prior studies, although this assertion is controversial. According to Yuan et al. [41], the diversity of mycobiomes is higher in healthy groups, with no difference in bacterial diversity observed. In addition, Liu et al. [42] reported no difference in the diversity of micro- and myco- biota, regardless of plant health. In light of this debate, interpretations of our results solely based on alpha diversity might not discern the nature of the association between gummosis and the microbiome; thus, the significant changes in alpha diversity we found were interpreted only after further analysis—namely, microbial classification, LEfSe, and network analysis.

Significant changes in taxonomic classification between HPB and PGB clarify the structural changes in bacterial communities. We discovered an intriguing characteristic of microbes that was drastically increased in PGB. Contrary to our assumptions, the microbial community in the bark of trees with gum disease was verified to contain far more microorganisms conducive to plant growth than pathogenic microbes. In addition, the majority of these microorganisms produce polysaccharides [43,44,45,46], which are one of the primary constituents of the gum that peach trees generate to defend themselves against insect pests and plant pathogens [47]. A previous study by Aitana Ares et al. [48] showed that *1174*-*901*-*12* (Rhizobiales), *Sphingomonas*, *Methylobacterium*-*Methylorubrum*, and *Pseudomonas* are commonly increased in plants infected with *Pseudomonas syringae* pv. *actinidiae*, suggesting the potential antagonistic properties of these bacteria against phytopathogenic fungi. This pilot study supports our hypothesis that host plants provide available resources to stimulate the development of beneficial endophytes in response to gummosis. In addition, the considerable surge in *Sphingomonas* following gummosis, regardless of the rhizosphere and endosphere, emphasizes the need for further research on the role of microbes in the environment.

We further identified discriminatory bacterial and fungal taxa between HPB and PGB. Five (*Pseudomonas*, *Methylobacterium*-*Methylorubrum*, *Sphingomonas*, *1174*-*901*-*12*, and *Catenibacterium*) out of 27 bacterial genera representing PGB selected with high LDA scores through LEfSe showed remarkable differences even in comparison to other 22 taxa, strengthening their potential as biomarker candidates. Among the fungi, *Alternaria* is the only taxon that differs statistically in both LEfSe and relative abundance comparisons between HPB and PGB. Surprisingly, we found that significantly increased fungal groups, including *Alternaria*, were predominantly composed of opportunistic plant pathogens, such as *Ascochyta*, *Botryosphaeria*, *Saccharomyces*, *Nectriaceae_NA*, *Trametes*, *and Valsaceae_NA* (Figure S2A). These findings suggest that fungi in a relative concentration of less than 1% can cause severe disease in the host, and the contradictory phenomenon of the simultaneous increase in probiotic bacteria and pathogenic fungi in PGB can be discussed from an ecological standpoint as a fierce competition between bacteria and fungi for survival in the complicated microflora.

As stated above, gummosis complicates the simple and stable microbial community in HPB; this symptom, dysbiosis, was demonstrated by the network analysis. Following gummosis, the number of nodes and edges increased double and tenfold, respectively, and the larger the number of nodes and edges, the greater the number of correlations. Our findings are inconsistent with previous claims that larger nodes, edges, and degrees of centralization indicate a stable network of healthy hosts [49]. This may be because a healthy standard bark network community has not been established due to a lack of active research on the bark microbiome, as well as the fact that bacteria and fungi that were dramatically altered in PGB were filtered out in prevalence and detection rates during the network construction. Nevertheless, these results will play an important role as fundamental reference data in enhancing our understanding of the response of bark microorganisms in additional gummosis research.

Despite the extensive analysis performed here, we acknowledge several limitations of this study. The current research was a cross-sectional study that analyzed the bark micro- and mycobiome following gummosis at random times, and it is essential to understand the precise plant–microbe interactions. Therefore, a longitudinal study monitoring changes in the microbial community is recommended to better understand the microbiome response to gummosis. In addition, the bacteria highlighted in PGB were investigated at the genus level; this leaves the possibility that the discovered genera are not consistent with the major pathogens—including *B. dothidea* or *p. syringae*. To overcome these problems, shotgun metagenome analysis must be carried out. Nevertheless, the novel results of the present study provide micro- and fungal-ecosystem structures in multiple environments and propose genera that are possibly linked to gummosis. Our findings contribute to a deeper comprehension of the microbial responses to gummosis and can be utilized to develop biomarkers for the early diagnosis of gummosis.

## 4. Materials and Methods

### 4.1. Rhizosphere Soil and Bark Sample Collection

**Figure 5:**
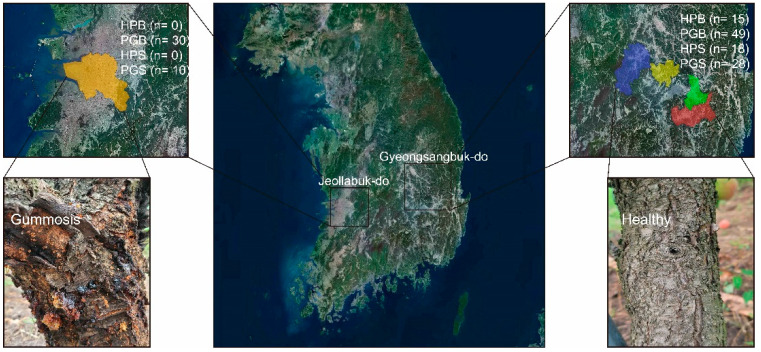
Geographical locations selected in this study. The number of bark and soil samples collected from the two provinces is displayed. Representative healthy bark and gummosis-infected bark are further shown.

Bark and soil samples of peach trees were collected from peach orchards in five different cities from April to September, 2021. In the five sampling sites, a total of 56 soil and 94 bark samples were collected, and the geographic location of each sampling site was visualized using QGIS 3 (Figure 5). Soil and bark samples were classified into two groups: the healthy group (HPS and HPB) and the peach gummosis group (PGS and PGB), according to the severity of gummosis reported by the farmers. Moreover, in order to collect bark with gummosis caused by the pathogen, bark without any physical wounds was collected. In brief, branches collected from the PG groups commonly contained resin and wilt. Consequently, the HP group included 18 HPS and 15 HPB samples, whereas the PG group yielded 38 PGS and 79 PGB samples. For soil and bark sampling, approximately 5 g of soil was collected from a depth of at least 10 cm from the topsoil and 1 cm from the tree root. Soil sample collection was carried out in the same way, regardless of the health status of the tree. HPB samples of uniform size were collected (length 2 cm, width 2 cm, depth 0.5 cm) using a sterilized carving knife, and PGB samples of uniform size were also collected after removing the resin from the surface of the gummosis area. The collected samples were stored in a 50 mL polypropylene tube and placed in a freezer at −70 °C until DNA extraction.

### 4.2. Microbial DNA Extraction

To extract total microbial DNA from bark and soil samples, bark samples were washed in 95% ethanol, 6% sodium hypochlorite, and 70% ethanol for 60 s, 6 min, and 30 s, respectively. We then cleaned the surface of the bark using sterile distilled water to remove microorganisms. This cleaning process was repeated six times. The cleaned tissue was cleaved to approximately 0.25 g using a sterilized razor blade and pulverized using a BioMasher-III (Optima Inc., Tokyo, Japan). DNA extraction from pretreated bark pieces and 0.25 g of soil was performed using a DNeasy PowerSoil Kit (Qiagen, Valencia, CA, USA), following the manufacturer’s instruction. The validity of the extracted DNA was confirmed through electrophoresis, and DNA was quantified using a Qubit fluorometer 2.0 (Waltham, MA, USA).

### 4.3. Library Preparation and High-throughput Sequencing

For the sequencing library preparation, V4–V5—the hypervariable region of the 16S rRNA gene—was amplified using a 515 F (5′-barcode-CGCTCTTCCGATCTGTGNCAGCMGCCGCGGTRA-3′) forward primer and a 907 R (5′-barcode-GTGCTCTTCCGATCCGYCWATTYHTTTRAGTTT-3′) reverse primer; the eukaryotic ITS2 region was amplified with the primer pairs ITS86F/ITS4R (ITS86F, 5′-ACACTC TTTCCCTACACGACGCTCTTCCGATCTGTGAATCATCGA ATCTTTGAA-3′, and ITS4R, 5′-GTGACTGGAGTTCAGACGTGTGCTCTTCCGATCTCCTCCGCTTATTGATATGC-3′). The specific PCR conditions used have been described in a previous study [50]. We then confirmed the library DNA size and quantity using gel electrophoresis and a Qubit fluorometer 2.0. Whole-library DNA was pooled in the same amounts and DNA purification was carried out using AMPure XP beads (Beckman Coulter, CA, USA). The libraries were sequenced using an Illumina MiSeq platform at the KNU NGS core facility (Daegu, Korea).

### 4.4. Bioinformatical Analysis

Raw sequencing data were processed using the Quantitative Insights into Microbial Ecology2 (QIIME2) pipeline (version 2021.4) [51]. In the raw amplicon data, the mean frequency was 16,740 reads for bacteria and 25,969 reads for fungi. Total numbers of sequence reads were manifested in individual samples using Illumina barcodes. After sequences with less than Q30 Phred quality scores and chimeric sequences were removed, amplicon sequence variants (ASVs) were obtained using DADA2 with default parameters [52]. The generated ASV IDs were assigned via the scikit-learn naive Bayes machine-learning classifier [53] based on the SILVA database for bacteria and the UNITE database for fungi, with a 99% cutoff (silva-138-99-515-806-nb-classifier.qza). Mitochondria, chloroplasts, and unassigned taxa were removed and the sequencing depth was rarefied to 5000 and 7000 reads for bacteria and fungi, respectively; the rarefaction curves were attached in the Supplementary Materials (Figure S3).

### 4.5. Computational and Statistical Analysis

To conduct the statistical analysis of the microbial community structure between HPB and PGB and HPS and PGS, multiple packages in R and GraphPad Prism 8.0.2 for Windows (GraphPad Software, San Diego, CA, USA) were employed with respect to both bacterial and fungal communities in the bark and soil. For a better understanding, we monitored various alpha diversity indices—observed, Shannon, and InvSimpson—using the “phyloseq” R package [54]. Analyses of beta diversity was computed based on the Bray–Curtis dissimilarity distance and the results were presented in a PCoA plot using the “vegan” R package [55].

Furthermore, we assessed the taxonomic distribution between the HP and PG groups at the phylum, family, and genus levels. Considering visibility, microbial classifications at the phylum and family levels were described in donut plots, and genera were separately displayed as a heatmap using the “pheatmap” R package. In the heatmap, genera with lower than 10% prevalence and 1% relative abundance were filtered out. To identify the genera that showed statistically significant differences between HP and PG in soil and bark, the Mann–Whitney test was implemented, and those groups with higher taxa abundances were marked with an asterisk.

LEfSe analysis, an estimation of specific taxa in which attributes differ significantly depending on the existence of gummosis, was performed using the Galaxy implementation (https://huttenhower.sph.harvard.edu/galaxy/ (accessed on 21 August 2022)). Only candidate taxa with LDA scores of 3.5 or greater were visualized.

Finally, we performed a network analysis to examine how genera and components of the microbial community in soil and bark samples interact with each other. Genera with a relative abundance of less than 1% and a prevalence of less than 50% within each group were excluded from the analysis. Using the “igraph” R package [56], we then generated networks of HPB, PGB, HPS, and PGS comprised of nodes, edges, transitivity, and density. Bacterial and fungal genera were displayed together in the network; the color and size of the nodes and the thickness of the edges indicated the phylum taxa, relative abundance, and correlation intensity, respectively. Correlation assessment was carried out based on Spearman’s rank correlation coefficients and only edges with a statistical *p*-value of less than 0.05 were visualized.

## 5. Conclusions

In this study, a large-scale characterization of endosphere and rhizosphere microbiomes was performed on peach bark and soil with or without symptoms of gummosis. We found that gummosis was associated with alterations in the bacterial and fungal communities in the bark. Distinctly increased levels of probiotic bacteria and pathogenic fungi are possibly associated with plant health. Despite the absence of a time point for tracing the relationship between gummosis severity and microbial alterations, our pilot study contributed to a better understanding of the microbial ecosystem in peach trees with acute plant disease. In conclusion, our findings propose the academic path of biomarker mining for use in the early diagnosis of gum disease.

## Data Availability

All of the raw 16S rRNA gene sequence data for this current study were deposited with the National Center for Biotechnology Information’s BioProject under the accession number PRJNA870061.

## Supplementary Materials

The following are available online at https://www.mdpi.com/article/10.3390/plants11212834/s1, Figure S1: Microbial dissimilarity between bark and soil according to gummosis; Figure S2: Genera with statistical differences between HP and PG; Figure S3: Rarefaction curves of Illumina MiSeq sequencing; Table S1: Microbial composition (%) of HPB, PGB, HPS, PGS, at phylum and family level.
