# Supplementary material for: Changes in Microbial Community Structure in Response to Gummosis in Peach Tree Bark
Source: Plants (Basel). 2022 Oct 25;11(21):2834. doi: 10.3390/plants11212834 (PMC9657254; doi:10.3390/plants11212834)
Supplement: Supplementary file 1 [file plants-11-02834-s001.zip › plants-1902214-supplementary materials.pdf]

Supplementary materials

Changes in microbial community structure in response to gummosis in peach tree bark

YoungJae Jo [1], Da-Ryung Jung [1], Tae-Hyung Park [2], Dokyung Lee [2], Min-Kyu Park [1], Kyeongmo Lim [1] and Jae-Ho Shin [1,2,3,*]

[1] Department of Applied Biosciences, Kyungpook National University, Daegu 41566, Republic of Korea
[2] Department of Integrative Biotechnology, Kyungpook National University, Daegu 41566, Republic of Korea
[3] NGS Core Facility, Kyungpook National University, Daehak-ro 80, Daegu 41566, Republic of Korea

* Correspondence: jhshin@knu.ac.kr; Tel.: +82-53-950-5716; Fax: +82-53-953-7233

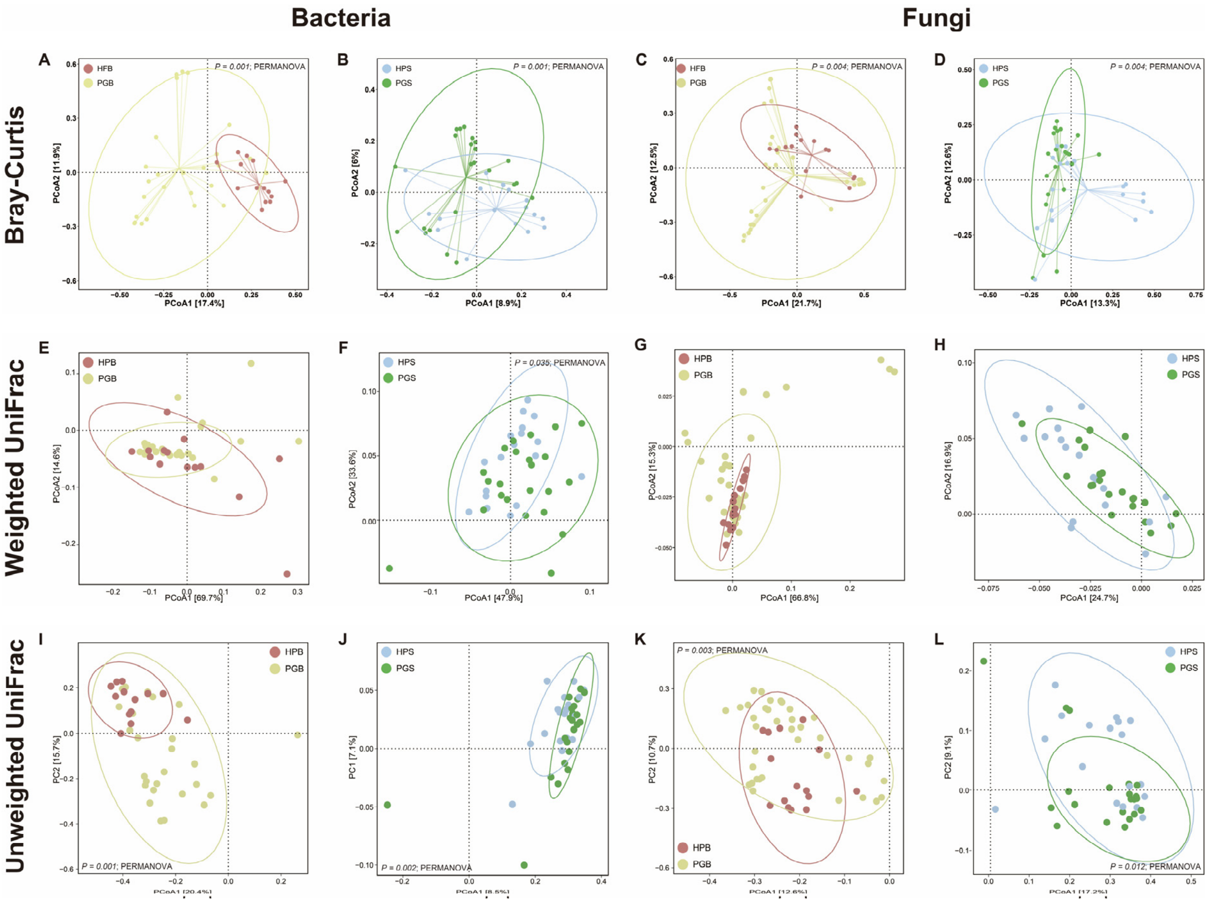

Figure S1. Microbial dissimilarity within bark and soil according to gummosis. All dissimilarity was computed using three strategies including Bray-Curtis (**A**-**D**), weighted UniFrac (**E**-**H**) and unweighted UniFrac (**I**-**L**). Each Permanova values were displayed in the individual plots.

Table S1. Microbial composition (%) of HPB, PGB, HPS, PGS, at phylum and family level.

Bacteria

Phylum	HPB	PGB	HPS	PGS
Acidobacteriota	2	3	12	16
Actionobacteriota	22	14	8	12
Crenarchaeota	2	3	10	7
Firmicutes	28	19	6	6
Proteobacteria	40	56	32	34
Other	6	5	24	25

Fungi

Phylum	HPB	PGB	HPS	PGS
Ascomycota	98	86	81	85
Basidiomycota	1	13	15	10
Kingdom_Fungi	1	1	2	2
Mortierellomycota	0	0	2	2
Other	0	0	0	1

Bacteria

Family	HPB	PGB	HPS	PGS
Beijerinckiaceae	1	15	1	2
Chitinophagaceae	0	0	4	6
Nitrososphaeraceae	2	3	10	7
Other	39	47	69	70
Paenibacillaceae	9	7	1	1
Propionibacteriaceae	16	5	0	0
Pseudomonadaceae	1	11	0	0
Rhodobacteraceae	30	3	0	0
Sphingomonadaceae	0	8	3	4
uncultured	1	0	6	5
Vicinamibacteraceae	1	0	6	5

Fungi

Family	HPB	PGB	HPS	PGS
Chaetomiaceae	23	4	8	12
Cladosporiaceae	5	5	3	4
Helotiaceae	3	7	2	3
Nectriaceae	12	12	21	26
Order_Microascales	2	0	5	3
Order_Onygenales	5	4	0	0
Other	44	46	53	44
Phylum_Ascomycota	5	7	2	1
Pseudeurotiaceae	2	2	5	6
Valsaceae	0	13	0	0

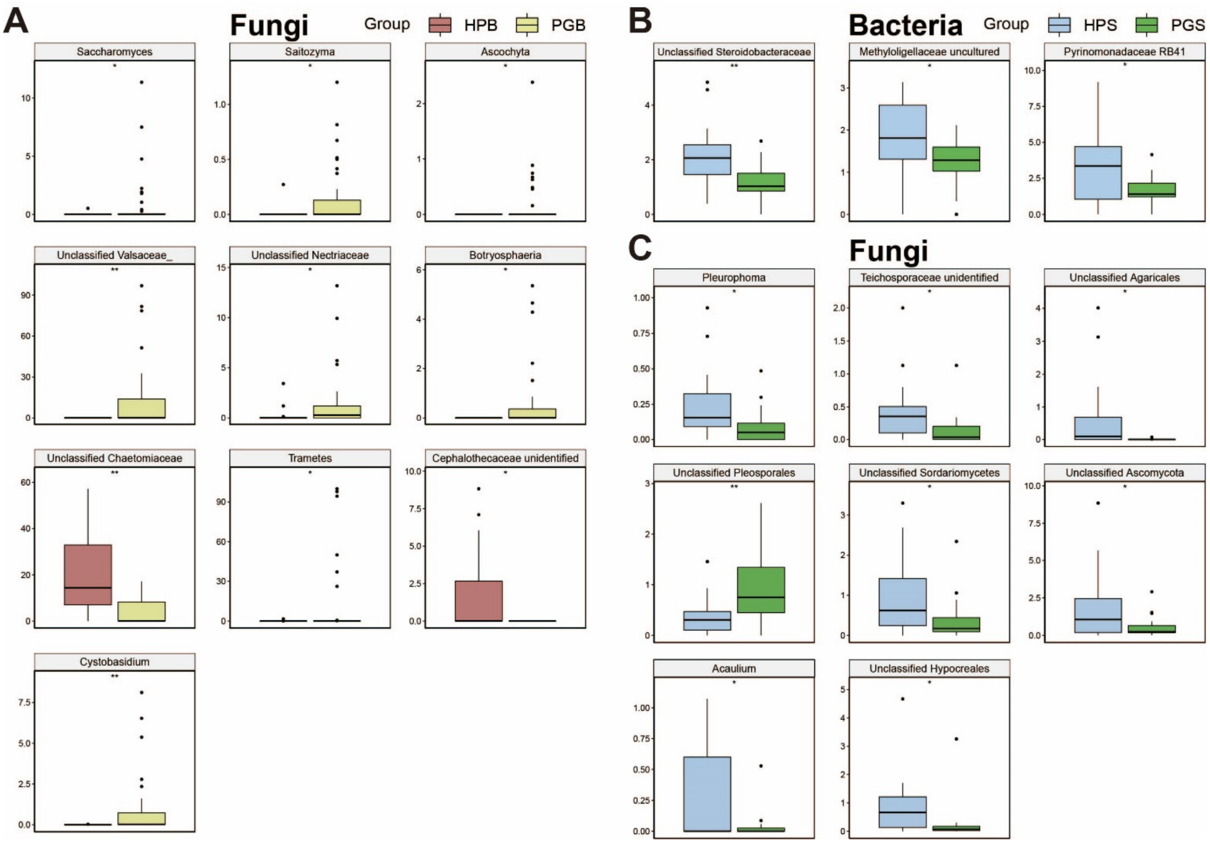

Figure S2. Genera with statistical difference between HP and PG. (**A**) Fungal genera between HFB and PGB. (**B**) and (**C**) Bacterial and fungal genera between HPS and PGS, respectively. All displayed genera show less than 10% of prevalence and 1% relative abundance in each groups. Percent (%) was adopted for the unit of bacterial concentration. Mann-Whitney U test was applied to assess statistical significance. * $p < 0.05$, ** $p < 0.01$.

A

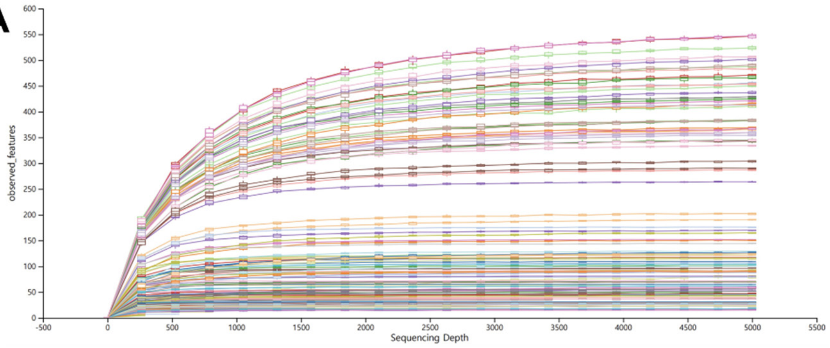

B

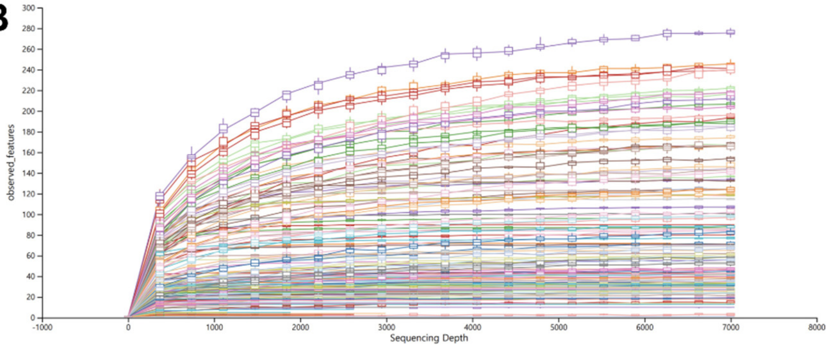

Figure S3. Rarefaction curves of Illumina MiSeq sequencing. (**A**) Rarefaction curve of bacterial sequencing with 5000 of sequencing depth (**B**) Rarefaction curve of fungal sequencing with 7000 of sequencing depth.
